# N-acetylcysteine Can Induce Massive Oxidative Stress, Resulting in Cell Death with Apoptotic Features in Human Leukemia Cells

**DOI:** 10.3390/ijms222312635

**Published:** 2021-11-23

**Authors:** Petr Mlejnek, Petr Dolezel, Eva Kriegova, Nikola Pastvova

**Affiliations:** 1 Department of Anatomy, Faculty of Medicine and Dentistry, Palacky University Olomouc, Hnevotinska 3, 77715 Olomouc, Czech Republic; p.dolezel@atlas.cz (P.D.); NikolaSkoupa@seznam.cz (N.P.); 2 Department of Immunology, Faculty of Medicine and Dentistry, Palacky University Olomouc, Hnevotinska 3, 77715 Olomouc, Czech Republic; eva.kriegova@email.cz

**Keywords:** HL-60 cells, U937 cells, oxidative stress, N-acetylcysteine, *NOX*, *SOD*, *MPO*

## Abstract

N-acetylcysteine (NAC), often used as an antioxidant-scavenging reactive oxygen species (ROS) in vitro, was recently shown to increase the cytotoxicity of other compounds through ROS-dependent and ROS-independent mechanisms. In this study, NAC itself was found to induce extensive ROS production in human leukemia HL-60 and U937 cells. The cytotoxicity depends on ROS-modulating enzyme expression. In HL-60 cells, NAC activated *NOX2* to produce superoxide (O_2_•^−^). Its subsequent conversion into H_2_O_2_ by superoxide dismutase 1 and 3 (*SOD1*, *SOD3*) and production of ClO^−^ from H_2_O_2_ by myeloperoxidase (*MPO*) was necessary for cell death induction. While the addition of extracellular *SOD* potentiated NAC-induced cell death, extracellular catalase (*CAT*) prevented cell death in HL-60 cells. The *MPO* inhibitor partially reduced the number of dying HL-60 cells. In U937 cells, the weak cytotoxicity of NAC is probably caused by lower expression of *NOX2*, *SOD1*, *SOD3*, and by the absence of MOP expression. However, even here, the addition of extracellular *SOD* induced cell death in U937 cells, and this effect could be reversed by extracellular *CAT*. NAC-induced cell death exhibited predominantly apoptotic features in both cell lines. Conclusions: NAC itself can induce extensive production of O_2_•^−^ in HL-60 and U937 cell lines. The fate of the cells then depends on the expression of enzymes that control the formation and conversion of ROS: *NOX*, *SOD*, and *MPO*. The mode of cell death in response to NAC treatment bears apoptotic and apoptotic-like features in both cell lines.

## 1. Introduction

N-acetylcysteine (NAC), despite its low bioavailability, has long been used in medicine for a wide range of conditions. It is a mucolytic agent in patients with a variety of respiratory illnesses, including cystic fibrosis. It is successfully used to treat paracetamol overdose, acute heavy metal poisoning, chemotherapy-induced toxicity, ischemia-reperfusion cardiac injury, diabetes, neuropsychiatric disorders, and many others. NAC also exhibits beneficial immune-modulation activity in patients with HIV. A detailed overview of NAC clinical applications can be found in the literature [1,2,3,4]. Its mechanism of action in clinics is mainly attributed to (a) its ability to reduce disulfide bonds and (b) to serve as a precursor of glutathione (GSH) synthesis [2,4,5].

In laboratory experiments, NAC is used as an efficient ROS scavenging agent that acts directly or indirectly through stimulation of GSH synthesis. However, the explanation that the protective effects of NAC lie in its ability to directly scavenge free radicals must be taken with caution. NAC serves as an efficient scavenger of hydroxyl radicals (•OH) and hypochlorous acid (HOCl), but it does not interact with superoxide (O_2_•^−^), the most important biologically relevant ROS, and it only interacts slowly with H_2_O_2_ [6,7]. Its chemopreventive properties are also attributed to its nucleophilicity, which blocks the cytotoxicity of reactive electrophiles [4,8]. While the ability of NAC to scavenge ROS directly or indirectly is often overestimated, its nucleophilic potential to neutralize the cytotoxic properties of many electrophiles is often overlooked. This is probably because the detection of the nucleophilic interaction of NAC with the electrophile requires a specialized analytical method that allows identification of the resulting NAC-electrophile adduct. For example, we recently demonstrated that NAC nucleophilicity rather than its ROS direct or indirect scavenging potential prevented cell death induced by geldanamycin, carbonyl cyanide 4-(trifluoromethoxy)phenylhydrazone, or 3,5-bis[(2-fluorophenyl)methylene]-4-piperidinone [9,10,11]. In addition, Georgiou-Siafis et al. demonstrated that NAC forms an adduct with hemin and that this reaction is the true protective mechanism of NAC against hemin cytotoxicity [12].

However, the biological effects of NAC are more complex. Several research groups have reported that NAC can even enhance cell death induced by other cytotoxic compounds under certain conditions. For example, NAC enhanced imatinib induced cell death via stimulation of NO production in human leukemia K562 [13] and it enhances fisetin induced apoptosis via ROS independent mechanism [14]. In addition, Zheng et al. reported that NAC interacts with Cu^2+^ to generate hydrogen peroxide, which induce cell death in cancer cells [15]. Importantly, we recently observed that low concentrations of NAC (0.5–1 mM) could enhance the cytotoxic effect of Cd^2+^ solely via the massive ROS production [16].

Here we report that NAC itself can induce extensive production of O_2_•^−^ in both promyelocytic leukemia HL-60 and histiocytic lymphoma U937 cell lines. We found that O_2_•^−^ alone does not induce cell death. Its cytotoxicity depends on the activity of ROS-modulating enzymes. The cytotoxicity of O_2_•^−^ can be affected either by the inherent expression of superoxide dismutase 1 and 3 (*SOD1*, *SOD3*) and myeloperoxidase (*MPO*), or by the addition of extracellular *SOD* and catalase (*CAT*) to the growth medium. In addition, we also studied the morphological and biochemical features of cell death induced by NAC treatment.

## 2. Results

### 2.1. Cytotoxic Effect of NAC in Human Leukemia Cells and ROS Production

**Figure 1:**
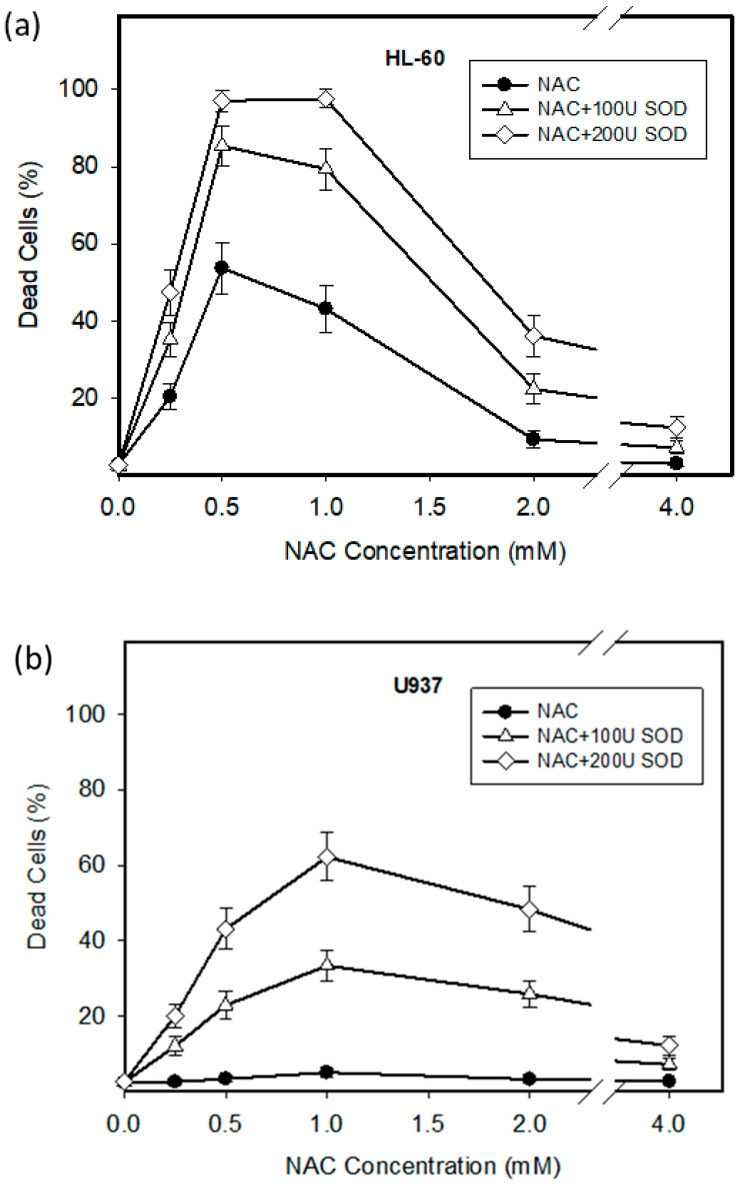
Effect of NAC on cell viability in HL-60 and U937 cells. Cells were treated with NAC or with NAC + *SOD*, as indicated. After 24 h incubation, the number of dead cells was determined using the trypan blue dye exclusion assay. (**a**) HL-60 cells. (**b**) U937 cells. The points represent the mean from three independent experiments with standard errors.

We observed that NAC, depending on concentration used, induced extensive loss of viability in HL-60 cells (Figure 1a). The greatest cytotoxic effect was observed for the 0.5–1 mM NAC concentrations (Figure 1a). Importantly, the cytotoxic effect of NAC could be potentiated by addition of extracellular *SOD* (Figure 1a). In contrast, U937 cells were only marginally affected by the same treatment (Figure 1b). However, the addition of extracellular *SOD* led to dramatic loss of cell viability (Figure 1b).

**Figure 2:**
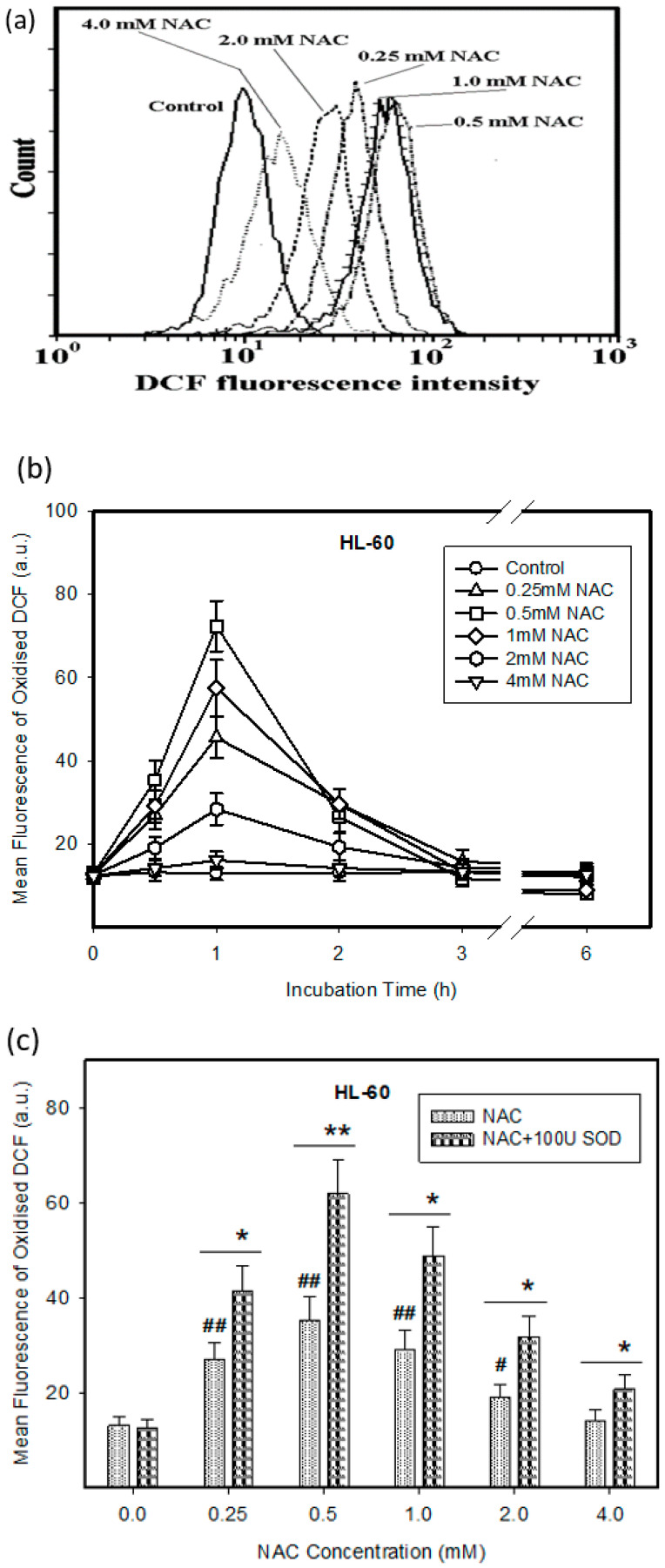

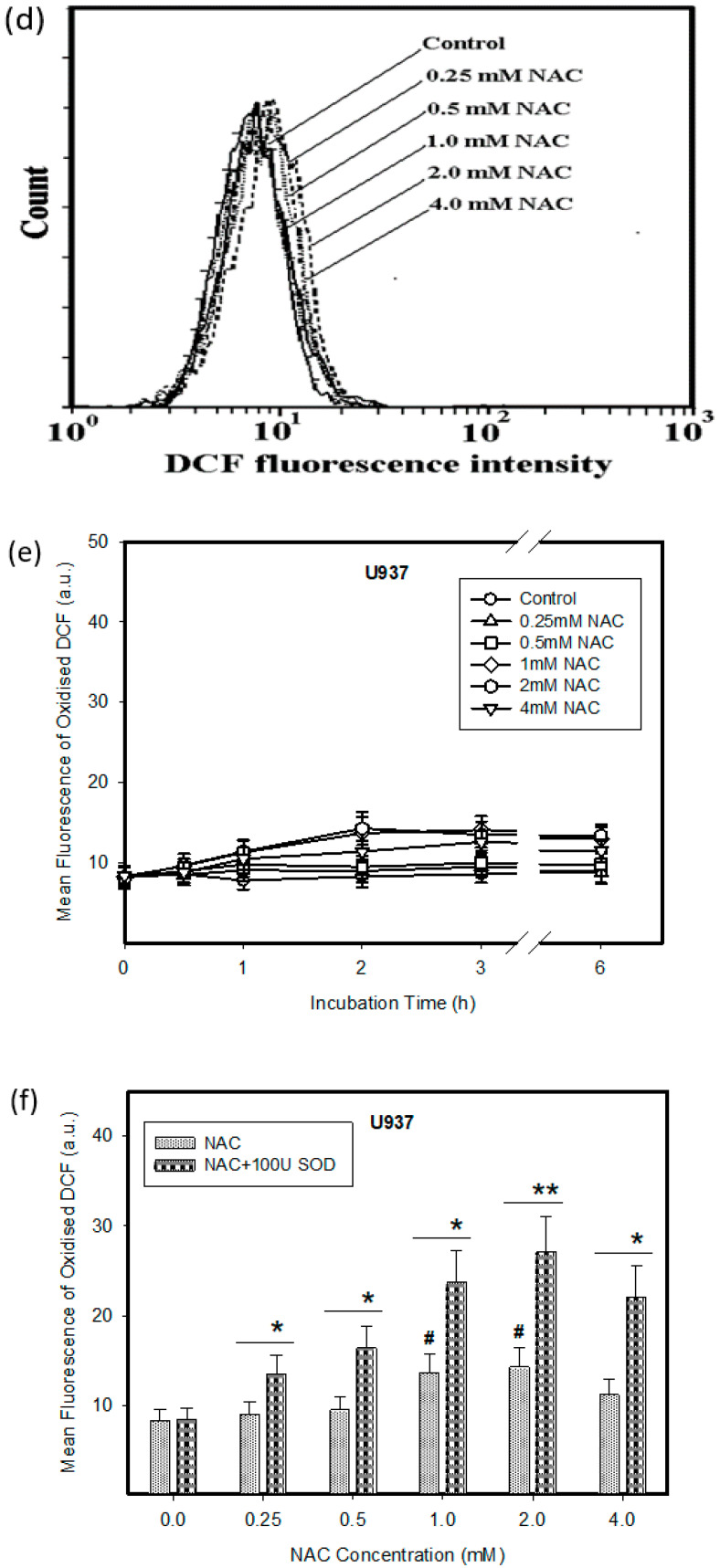
Effect of NAC on ROS production in HL-60 and U937 cells. Cells were treated with NAC as indicated. After the indicated period, the incubation cells were stained with H_2_DCF-DA and fluorescence intensity was analyzed using flow cytometry. (**a**) Effect of NAC on ROS production in HL-60 cells (a typical analysis). (**b**) Time course of ROS production in NAC treated HL-60 cells. (**c**) Effect of *SOD* on ROS production in NAC treated HL-60 cells. Points represent mean value from three independent experiments with standard errors. (**d**) Effect of NAC on ROS production in U937 cells (a typical analysis). (**e**) Time course of ROS production in NAC treated U937 cells. (**f**) Effect of *SOD* on ROS production in NAC treated U937 cells. Points represent mean value from three independent experiments with standard errors. **^#^**/**^##^** denotes significant/very significant difference in the mean fluorescence of oxidized DCF (*p* < 0.05/*p* < 0.01) between the NAC treated and untreated (control) cells. */** denotes significant/very significant difference in the mean fluorescence of oxidized DCF (*p* < 0.05/*p* < 0.01) between the indicated treatments.

We further found that the treatment of HL-60 cells with NAC was accompanied by the extensive intracellular oxidation of H_2_DCF-DA to DCF indicating H_2_O_2_ production (Figure 2a,b). Extracellular *SOD* enhanced H_2_O_2_ production in NAC treated HL-60 cells (Figure 2c). In contrast, only marginal H_2_O_2_ production was observed in response to NAC in U937 cells (Figure 2d,e). However, even here, the addition of extracellular *SOD* significantly increased H_2_O_2_ production (Figure 2f).

**Figure 3:**
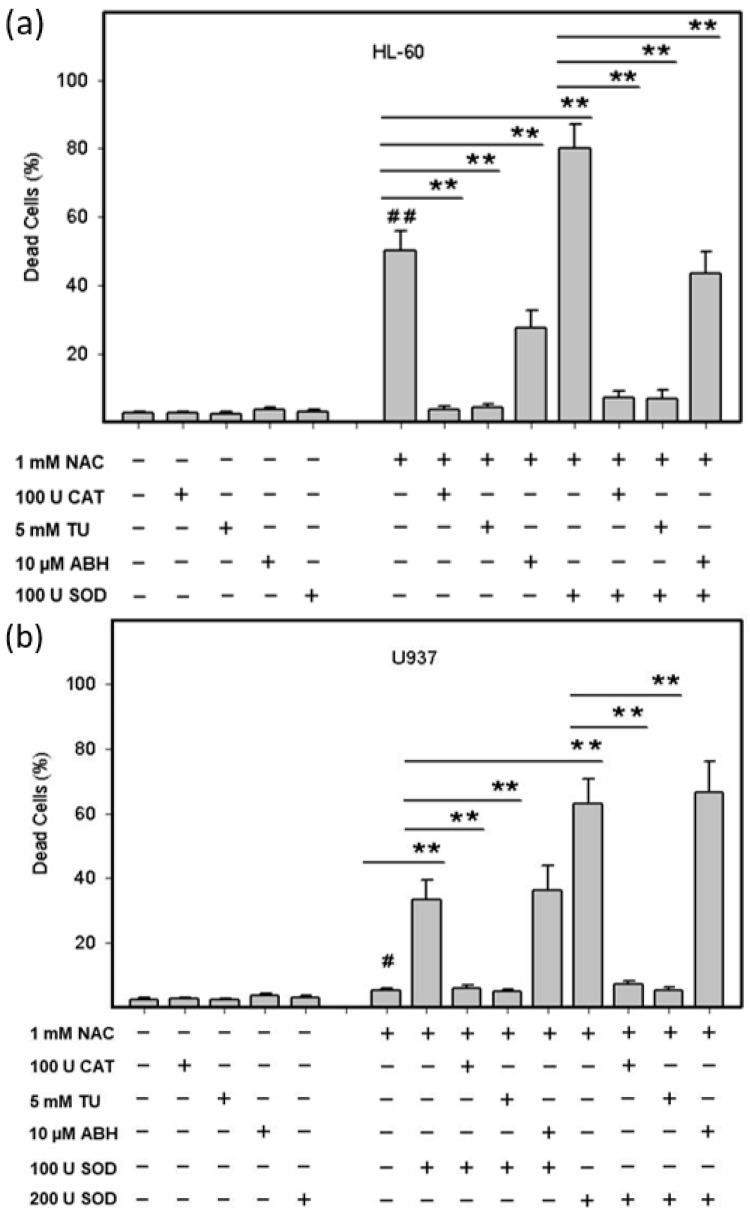
Effect of ROS modulators on viability of NAC treated HL-60 and U937 cells. Cells were treated with NAC or with NAC in combinations with ROS modulators as indicated. After 24 h incubation, the number of dead cells was determined using the trypan blue dye exclusion assay. (**a**) HL-60 cells. (**b**) U937 cells. Columns represent the mean from three independent experiments with standard errors. **^#^**/**^##^** denotes significant/very significant difference in the number of dead cells (*p* < 0.05/*p* < 0.01) between the NAC treated and untreated (control) cells. ** denotes very significant difference in the number of dead cells (*p* < 0.01) between the indicated treatments.

Next, we addressed the question whether the ROS production was actually responsible for the cell death in the HL-60 and U937 cells. For this reason, we studied the effect of thiourea (TU), which serves as a superior radical scavenger for ROS [17], extracellular *CAT*, and 4-aminobenzoic acid hydrazide (ABH), a *MPO* inhibitor, on cell death induced by NAC or by NAC in combination with extracellular *SOD* in both cell lines. Addition of TU or *CAT* to growth medium abrogated cell death in HL-60 cells treated with NAC or with NAC in combination with *SOD* (Figure 3a). TU and *CAT* also prevented cell death in U937 cells treated with NAC in combination with *SOD* (Figure 3b). In addition, ABH reduced the cytotoxic effect of NAC or NAC in combination with *SOD* by approximately half in HL-60 cells (Figure 3a). In contrast, ABH did not affect cell death induced by NAC in combination with *SOD* in U937 cells (Figure 3b).

**Figure 4:**
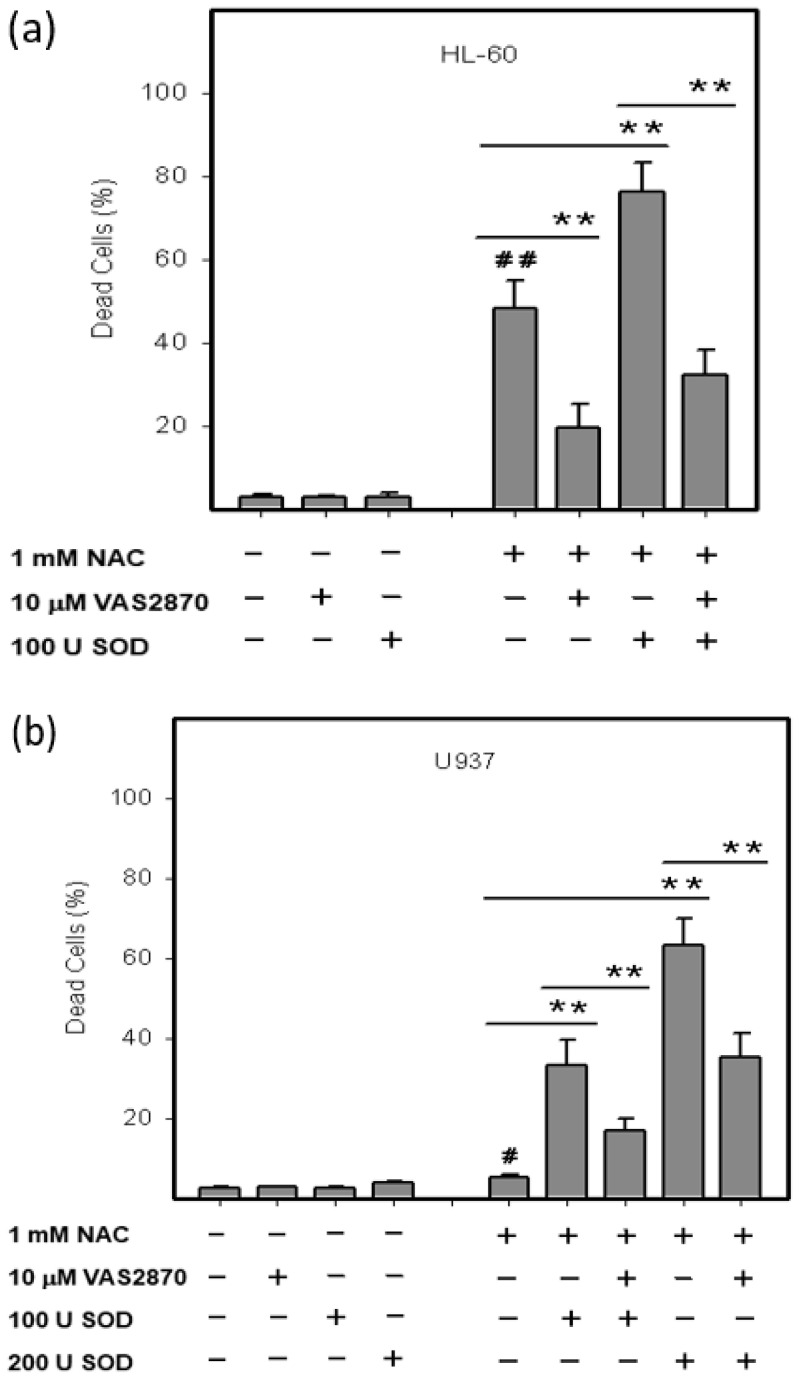
Effect of VAS2870 on viability of NAC treated HL-60 and U937 cells. Cells were treated with NAC in combinations with VAS2870 or with NAC in combinations with *SOD* and VAS2870 as indicated. After 24 h incubation, the number of dead cells was determined using the trypan blue dye exclusion assay. (**a**) HL-60 cells. (**b**) U937 cells. Columns represent the mean from three independent experiments with standard errors. ^#^/^##^ denotes significant/very significant difference in the number of dead cells (*p* < 0.05/*p* < 0.01) between the NAC treated and untreated (control) cells. ** denotes very significant difference in the number of dead cells (*p* < 0.01) between the indicated treatments.

Experiments with extracellular *SOD* have clearly suggested that the initial source of ROS was O_2_•^−^ the source of which could be various *NOX(s)*. Therefore, we used VAS2870, a cell permeable inhibitor of *NOX* [18]. Figure 4a shows that VAS2870 had significant cytoprotective effect in HL-60 cells treated with NAC or NAC in combination with extracellular *SOD*. VAS2870 worked similarly in NAC-treated U937 cells in combination with extracellular *SOD* (Figure 4b). These results clearly indicated that the source of O_2_•^−^ was a *NOX*.

### 2.2. Expression of ROS Related Genes

**Figure 5:**
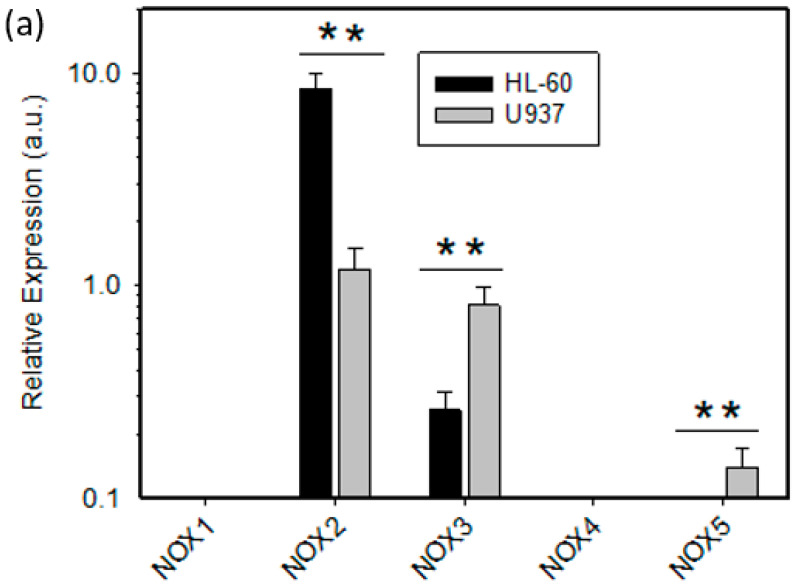

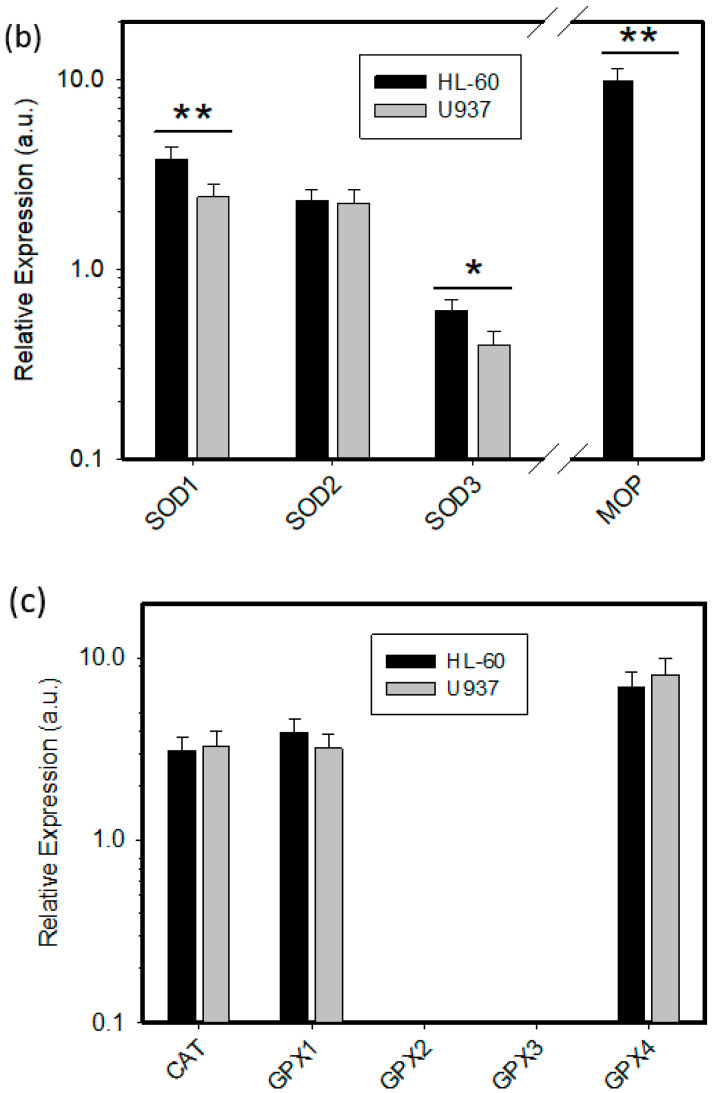
Analysis of the mRNA expression of ROS-related genes in HL-60 and U937 cells. (**a**) Expression analysis of *NOX1*–*NOX5* genes. (**b**) Expression analysis of *SOD1*–*SOD3,* and *MPO* genes. (**c**) Expression analysis of *CAT* and *GPX1*–*GPX4* genes. Results represent the mean values of four independent experiments with standard errors. */** denotes significant/very significant difference in the relative expression of appropriate ROS-related gene (*p* < 0.05/*p* < 0.01) between HL-60 and U937 cells.

We further analyzed the mRNA expression of ROS related genes *NOX1-5*, *SOD1-3*, *GPX1-4*, *CAT*, and *MPO* to further refine the contribution of individual genes to the cytotoxic effects of NAC in HL-60 and U937 cells. First, we studied expression of *NOX* genes, which we considered as primary source of ROS in the studied experimental system. Our results revealed that *NOX1* and *NOX4* were not expressed either in HL-60 or in U937 cells (Figure 5a). HL-60 cells exhibited 6–8 times higher expression of *NOX2* than U937 cells (Figure 5a). In contrast, *NOX3* expression was 3–4 times lower in HL-60 cells compared to U937 cells (Figure 5a). Low expression of *NOX5* was found only in U937 cells (Figure 5a). Next we studied the expression of *SOD* genes, whose products are responsible for the conversion of O_2_•^−^ to H_2_O_2_. Expression of *SOD1* was 1.5–2 times higher in HL-60 cells than in U937 cells (Figure 5b). Expression of *SOD2* was approximately the same in both cell lines (Figure 5b). *SOD3* was expressed approximately 1.5 times higher in HL-60 cells than in U937 cells, however, the overall expression was low (Figure 5b). Genes *CAT*, *GPX1*, and *GPX4,* whose products are involved in the breakdown of H_2_O_2_, and phospholipid hydroperoxide reduction, were expressed at similar levels in both cell lines (Figure 5c). No *GPX2* and *GPX3* expression was detected in either cell line (Figure 5c). *MPO* was expressed exclusively in HL-60 cells (Figure 5b).

### 2.3. Origin and Interconversion of ROS and Their Relation to Cell Death

**Figure 6:**
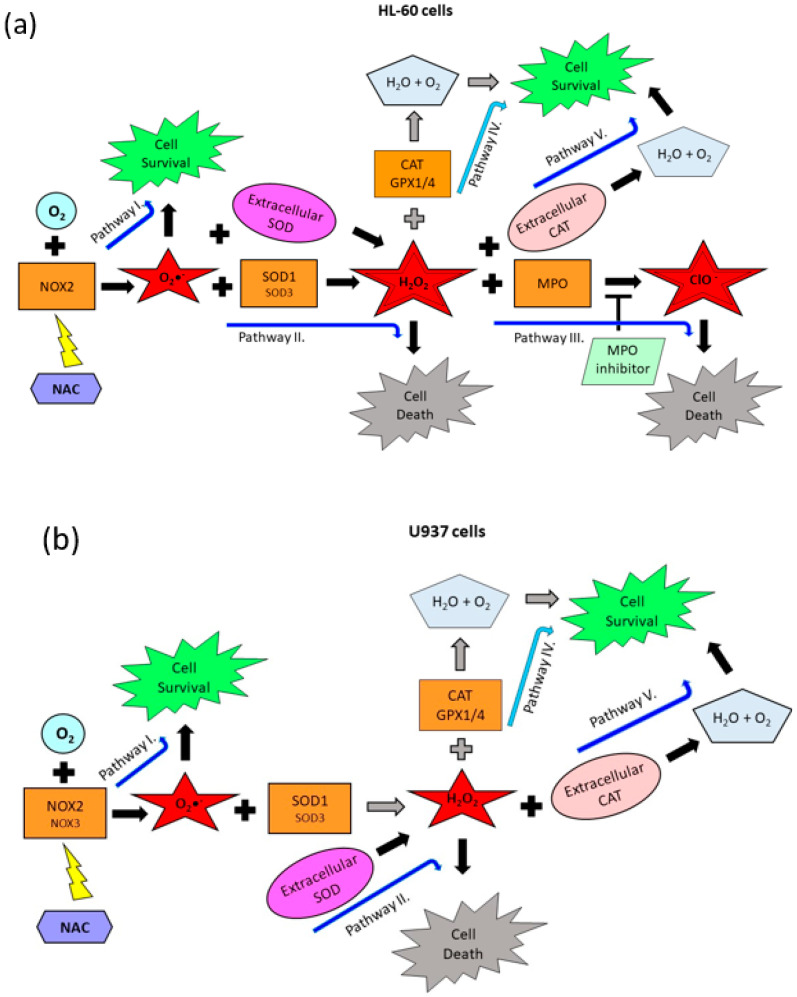
Identification of genes that are responsible for ROS production and their contribution to cell death induction. (**a**) Proposed scheme for HL-60 cells. Massive production of O_2_•^−^ by *NOX2* was not sufficient to induce cell death (Pathway I). Higher expression of *SOD1* and *SOD3* in HL-60 cells led to the induction of cell death (Pathway II). Inherent expression of *CAT*, *GPX4*, and *GPX4* failed to prevent H_2_O_2_-induced cell death (Pathway IV). Conversion of H_2_O_2_ to ClO^−^ by inherent *MPO* led to the cell death induction (Pathway III). Extracellular *SOD* increased the number of dying cells (it strengthens the Pathway II and III). Extracellular *CAT* completely prevented cell death (Pathway V) (**b**) Proposed scheme for U937 cells. Elevated production of O_2_•^−^ by *NOX2(3)* was not sufficient to induce cell death (Pathway I). Either lower expression of *SOD1* and *SOD3* in U937 cells failed to induce cell death (Pathway II) or inherent expression of *CAT*, *GPX4*, and *GPX4* failed to prevent H_2_O_2_-induced cell death (Pathway IV). While extracellular *SOD* induced cell death (Pathway II), extracellular *CAT* completely prevented *SOD*-induced cell death (Pathway V).

Based on the analysis of the expression of ROS modulating genes and the results above, we proposed a hypothetical scheme with identification of biochemical pathways responsible for the origin, transformation and/or degradation of individual ROS and their possible contribution to cell death or cell survival in both cell lines. (Figure 6). These observations collectively suggested that the primary ROS induced by NAC was superoxide radical O_2_•^−^ that was produced by *NOX(s)* close to the cell membrane or extracellularly since VAS2870 and extracellular *SOD* and *CAT* can significantly modulate the viability in both cell lines (Figure 1, Figure 2, Figure 3, Figure 4, Figure 5 and Figure 6). The probable producer of superoxide is *NOX2* in HL-60 cells and *NOX2/3* in U937 cells (Figure 5 and Figure 6). Interestingly, even massive production of O_2_•^−^ by *NOX2/3* was not sufficient to induce cell death in either cell line (Figure 6—Pathway I) H_2_O_2_, a product of SOD1/3, together with ClO^−^, a product of *MPO*, were probably the most important cell death related ROS in HL-60 cells (Figure 3a, Figure 5, and Figure 6a—Pathway II and Pathway III). Inherent expression of *CAT* and *GPX1/4* failed to prevent cell death in HL-60 cells (Figure 5 and Figure 6a—Pathway IV). In contrast, H_2_O_2_ was probably the most important cell death related ROS in U937 cells (Figure 3b, Figure 5, and Figure 6b—Pathway II). However, cytotoxic levels of H_2_O_2_ can be achieved only by extracellular *SOD* but not by SOD1/3. This is due either to the low activity of SOD1/3 or due to the protective effect of *CAT* plus *GPX1/4* (Figure 5 and Figure 6b—Pathway IV).

### 2.4. Mode of Cell Death Induced by NAC

**Figure 7:**
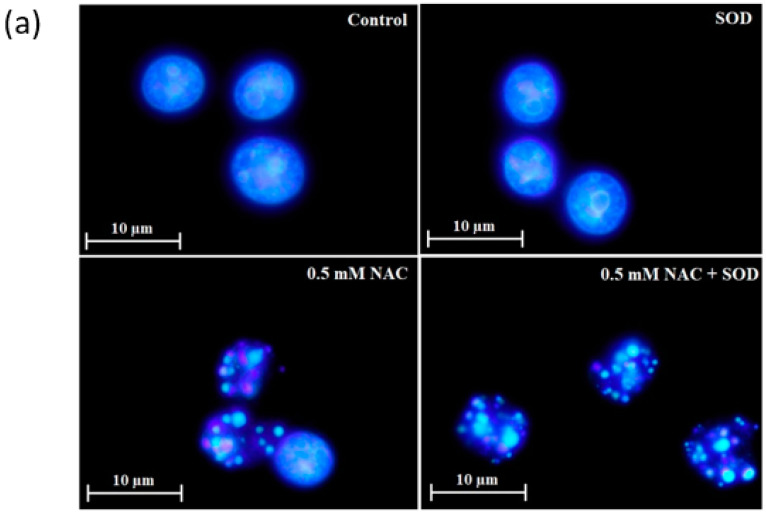

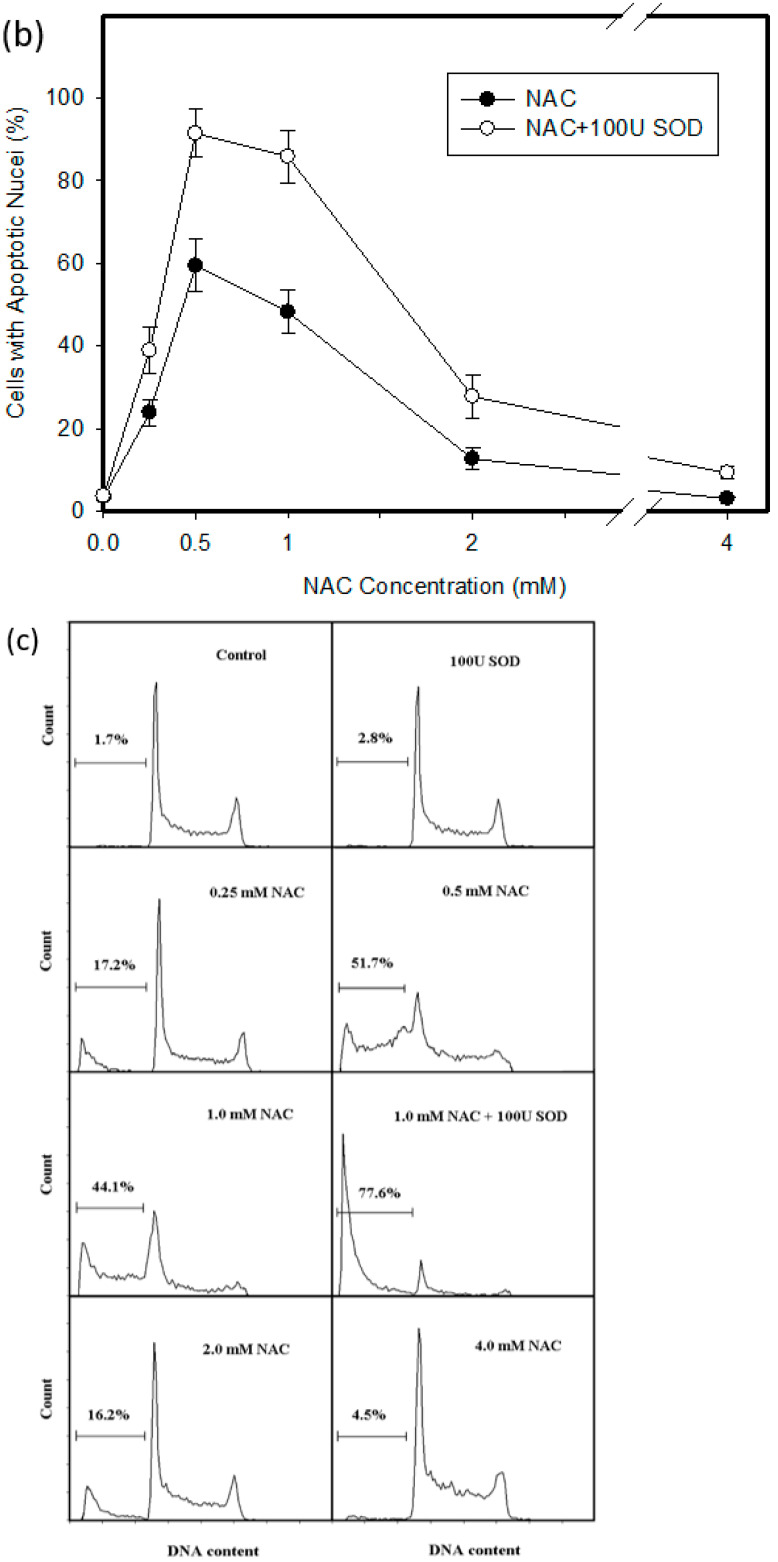

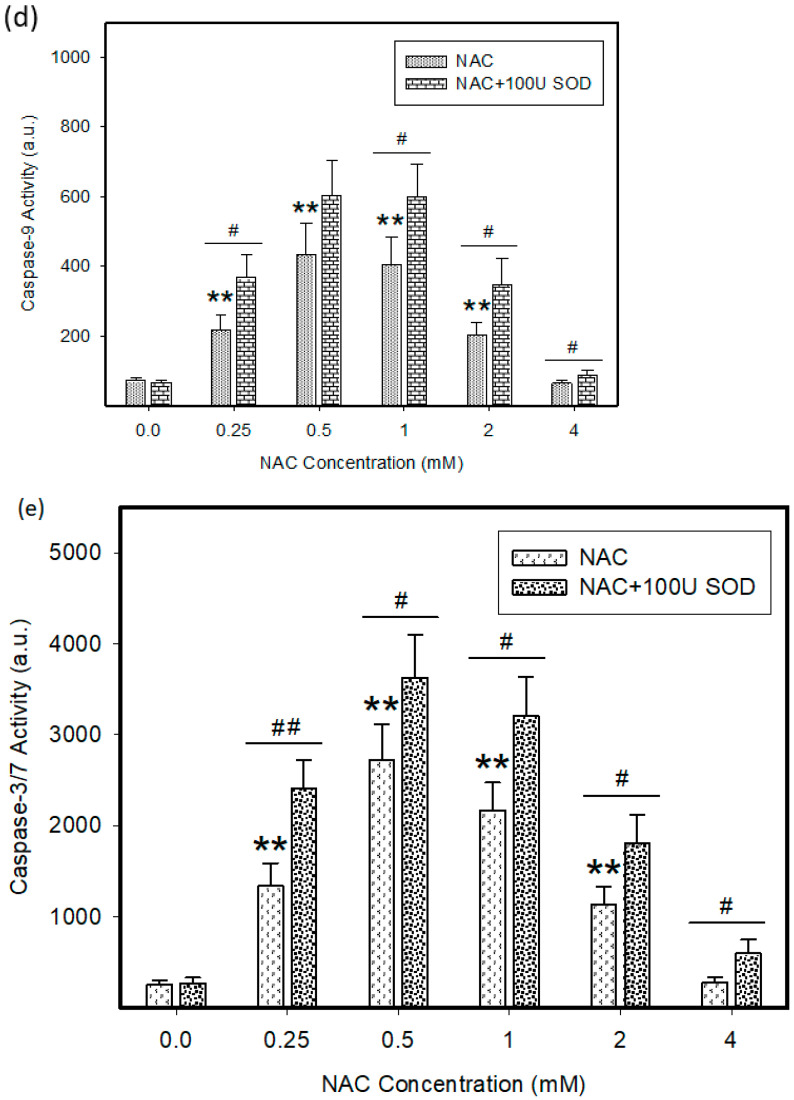
Effect of NAC on cell death features in HL-60 cells. Cells were treated with NAC or with NAC in combination in *SOD* as indicated. After 24 h incubation, cell death features were analyzed. (**a**) Effect on nuclear morphology. Pictures represent typical examples. (**b**) Effect on nuclear morphology—a quantitative analysis. Points represent mean from three independent experiments with standard deviations. (**c**) Effect on DNA fragmentation (hypodiploid DNA content). Histograms represent typical examples. (**d**) Effect on caspase-9 activation. Points represent mean from three independent experiments with standard errors. (**e**) Effect on caspase-3/7 activation. Points represent mean from three independent experiments with standard errors. ** denotes very significant difference in the caspase activity (*p* < 0.05/*p* < 0.01) between the NAC treated and untreated (control) cells. **^#^**/**^##^** denotes significant/very significant difference in the caspase activity (*p* < 0.01) between the indicated treatments.

**Figure 8:**
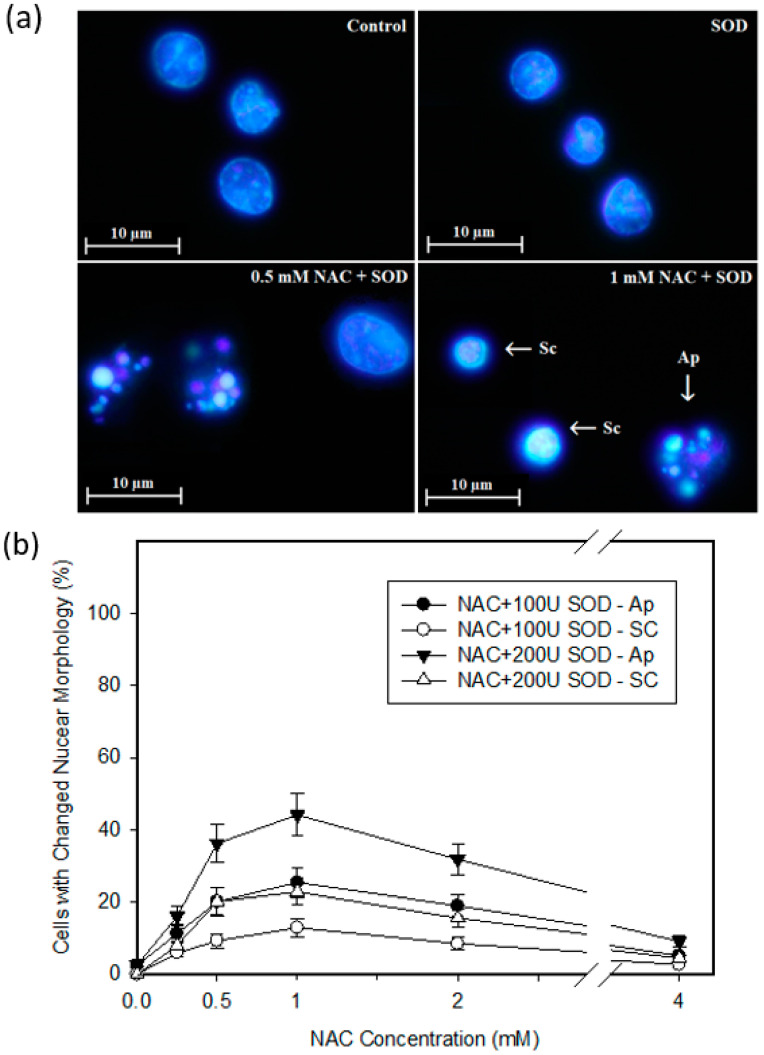

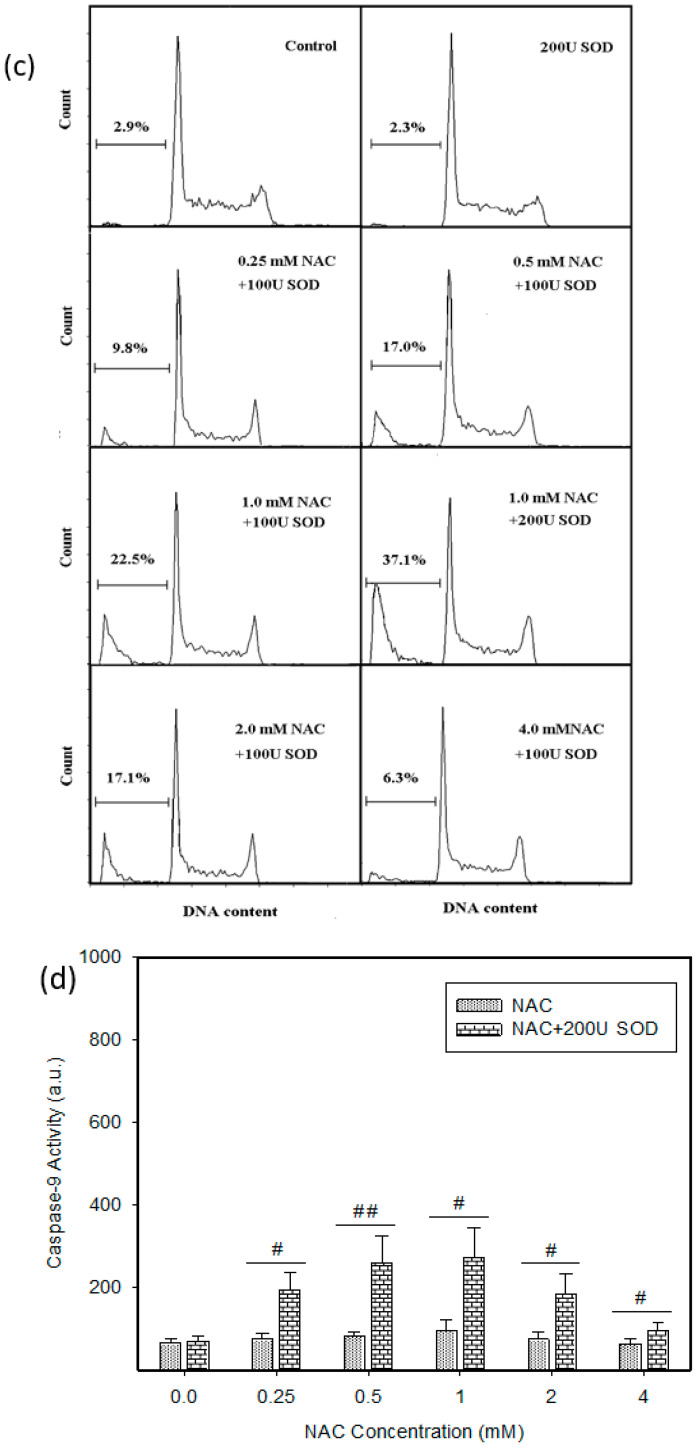

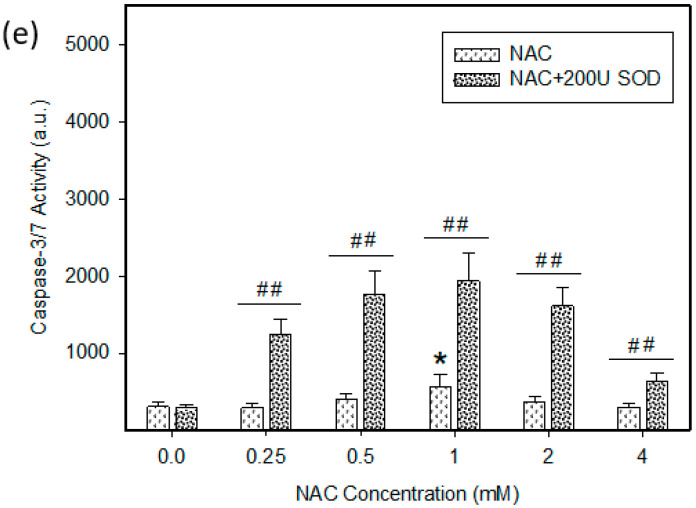
Effect of NAC on cell death features in U937 cells. Cells were treated with NAC or with NAC in combination in *SOD* as indicated. After 24 h incubation, cell death features were analyzed. (**a**) Effect on nuclear morphology. Pictures represent typical examples. (**b**) Effect on nuclear morphology—a quantitative analysis. Points represent mean from three independent experiments with standard errors. (**c**) Effect on DNA fragmentation (hypodiploid DNA content). Histograms represent typical examples. (**d**) Effect on caspase-9 activation. Points represent mean from three independent experiments with standard errors. (**e**) Effect on caspase-3/7 activation. Points represent mean from three independent experiments with standard errors. * denotes significant difference in the caspase activity (*p* < 0.05) between the NAC treated and untreated (control) cells. **^#^**/**^##^** denotes significant/very significant difference in the caspase activity (*p* < 0.05/*p* < 0.01) between the indicated treatments.

Next, we studied the mode of cell death elicited by NAC. We observed that dying HL-60 cells exhibited morphological and biochemical features of apoptosis, including chromatin condensation (Figure 7a,b), DNA fragmentation (Figure 7c), caspase-9 (Figure 7e), and caspase-3/7 activation (Figure 7f). Importantly, addition of *SOD* increased the number of dead cells with typical apoptotic hallmarks (Figure 7). In U937 cells treated with NAC in combination with *SOD* was an increase in the number of dead cells with predominantly morphological and biochemical features of apoptosis (Figure 8). Importantly, in some dead cells we observed small condensed nuclei (Figure 8a,b), a morphology which is typical for cells undergoing apoptotic program suppressed by pan-specific caspase inhibitor Z-VAD-FMK [19,20]. No cells with swollen nuclei typical for necrosis were found (not shown).

## 3. Discussion

Although redox reactions are currently understood in the broader context of their complexity, we still view a number of agents as typical ROS scavengers (formerly antioxidants). One example is NAC, whose protective effects are attributed to its ability to scavenge ROS directly or indirectly through stimulation of intracellular GSH synthesis in laboratory experiments. Its protective effects are also ascribed to its nucleophilicity, which blocks the cytotoxicity of reactive electrophiles [5,8]. Importantly, some recently published studies show that in certain situations NAC can potentiate the cytotoxic effects of some substances, via the induction of massive ROS or NO production [13,14,15,16]. However, to our knowledge, NAC alone has never been described as a direct inducer of ROS, which, depending on the concentration of NAC used and activity of ROS-modulating enzymes, may even induce cell death in human leukemia HL-60 and U937 cells. Interestingly, NAC has a maximal cytotoxic effect at low millimolar concentrations (Figure 1) at which concentrations it otherwise has cytoprotective effects in most laboratory experiments [5,8]. The cytotoxic effects of NAC decrease at higher and lower concentrations (Figure 1). Unfortunately, for this the “bell like shape” of the NAC cytotoxicity curve, we have no adequate explanation.

Our results show that NAC itself induces extensive production of O_2_•^−^ in both, HL-60 and U937 cells, albeit, the production of superoxide is lower in U937 cells (Figure 1, Figure 2, Figure 3, Figure 4, Figure 5 and Figure 6). The source of O_2_•^−^ is *NOX(s)* in both cell lines, as judged from the effect of VAS2870 (Figure 4). Considering the very low stability of superoxide in aqueous solutions [21] and the fact that in our experimental system the conversion of O2•^−^ to H_2_O_2_ can be modulated by the addition of extracellular *SOD* (Figure 1, Figure 2 and Figure 3), it is reasonable to assume that its production takes place near the cell surface in both cell lines.

Detailed analysis revealed that the main source of O_2_•^−^ production is probably *NOX2* in HL-60 cells (Figure 5a and Figure 6a). Other *NOXs* do not appear to play any significant role in superoxide production because they are either not expressed (*NOX1*, *NOX4*, *NOX5*) or their expression is very low compared to *NOX2* (*NOX3)* (Figure 5a). Our data are thus in good agreement with the findings of other authors on the expression, function, and localization of *NOX2* [22,23].

The situation is different in U937 cells. Although *NOX2* is the most expressed type of *NOX* gene, its expression is 6–8 times lower than that of HL-60 cells (Figure 5a). Due to the relatively high expression of *NOX3*, which is 3–4 times higher than in HL-60 cells (Figure 5a), we hypothesize that both *NOX2* and *NOX3* may be involved in superoxide production in U937 cells (Figure 6b). Since there is very low expression of *NOX5*, we assume it does not contribute to superoxide formation as is the case with *NOX1* and *NOX4,* which are not expressed (Figure 5a). However, the specific contribution of *NOX2* and *NOX3* to superoxide formation in U937 cells is not clear and this issue needs further study.

The possibility that production of O_2_•^−^ was mediated by *NOX* isoforms, DUOX1 and DUOX2, was considered to be improbable, since our results suggest its cell surface close production (see above) and these enzymes tend to be retained deep inside the cells in the endoplasmatic reticulum (ER) in such a way that their activity can be measured only on plasma membrane disruption [22]. For this reason, we did not analyze their expression in the studied cell lines.

However, the O_2_•^−^ itself does not seem to be responsible for the induction of cell death in our experimental system, as its subsequent conversion to H_2_O_2_ was the necessary condition for manifesting cytotoxic effects in both cell lines (Figure 3, Figure 4, Figure 5 and Figure 6). Thus, our conclusions are in accordance with the findings of other authors who demonstrated that O_2_•^−^ itself has direct relationship to autophagy [24]; however, its conversion to either other ROS, such as the •OH radical via the Fenton reaction, or to peroxynitrite (ONOO^−^) via reaction with nitric oxide, causes damage to cellular macromolecules and lipids, which can end in cell death [25].

In HL-60 cells, O_2_•^−^ is likely converted to H_2_O_2_ by endogenous *SOD1* and *SOD3* (Figure 5b and Figure 6a). H_2_O_2_ is further partly converted to ClO^−^ by endogenous MOP (Figure 5b and Figure 6a). The conclusion that the conversion of H_2_O_2_ to ClO^−^ is partial, stems from the finding that the protective effect of *MPO* inhibitor is also partial (Figure 3a). Collectively, these findings suggest that both ROS species, H_2_O_2_ and ClO^−^, contribute to the loss of cell viability in HL-60 cells (Figure 3a, Figure 5b, and Figure 6a). We can further speculate about the possible role of the •OH radical in cell death induction since the Fenton reaction can give rise to this radical from H_2_O_2_ [26]. The •OH radical rather than H_2_O_2_ may be the specific ROS directly involved in the death of HL-60 cells. However, this question has yet to be examined experimentally. Surprisingly, the antioxidant enzymes involved in H_2_O_2_ and phospholipid hydroperoxide reduction, *GPX4*, *CAT*, and *GPX4*, although highly expressed endogenously, fail to protect HL-60 cells from NAC induced cell death (Figure 1, Figure 2 and Figure 3, Figure 5c and Figure 6a). The inability of *CAT*, *GPX4*, and *GPX4* to efficiently degrade H_2_O_2_ and phospholipid hydroperoxide may be due to spatial differences in their expression and the site(s) with highest concentration.

In contrast, probably the lower expression of *SOD1* and *SOD3* in combination with high endogenous expression of *GPX4*, *GPX4*, and *CAT* prevent the massive production of H_2_O_2_ from inducing cell death in U937 cells (Figure 3b, Figure 5b,c, and Figure 6b). However, the question of whether and to what extent endogenous *GPX4*, *GPX4*, and *CAT* expression provides protection against the cytotoxic effects of peroxides cannot yet be answered. This topic must be further studied. Only addition of extracellular *SOD* induces extensive cell death in NAC treated U937 cells (Figure 1b, Figure 3b, and Figure 8). Even here, it is uncertain whether the ROS directly involved in cell death induction is the H_2_O_2_ or •OH radical. However, recent research has shown that U937 cells actually convert O_2_•^−^ via H_2_O_2_ to •OH radical [27]. Therefore, it is very likely that cell death is induced by •OH radical and not H_2_O_2_. However, further research will provide the exact answer.

The experimental results of this study are summarized in Figure 6, which provides detailed hypothetical biochemical pathways for NAC to exert its cytotoxic effects through particular ROS in HL-60 and U937 cells.

Our results further suggest that NAC induced cell death with morphological and biochemical apoptotic hallmarks in HL-60 cells (Figure 7). We assume that the intrinsic pathway is activated as judged from elevated activity of caspase-9 (Figure 7d). The enhanced cytotoxic effect of NAC by the addition of extracellular *SOD* increases the number of dead cells without changing the type of cell death in HL-60 cells (Figure 7). The situation is slightly different for U937 cells (Figure 8). Here, too, the addition of extracellular *SOD* causes cell death with predominantly apoptotic features (Figure 8). However, some dead cells do not have typical morphological apoptotic features. Instead of apoptotic bodies, we can observe small condense nuclei (Figure 8a,b). We have previously described this morphologically somewhat unusual type of cell death in human leukemia cells [16,19,20]. Such nuclear morphology was found in experimental systems where the cells underwent an apoptotic program, and, at the same time, caspase activity was inhibited either by a pan-specific caspase inhibitor [19,20] or by cadmium [16]. Importantly, caspase-9 and caspase-3/7 activity is evidently lower in dying U937 than HL-60 cells (compare Figure 1a,b, Figure 7d and Figure 8d). Based on published results, it can be assumed that the H_2_O_2_ formed above a certain concentration may completely inhibit caspase activity [28].

## 4. Materials and Methods

### 4.1. Cell Culture

Human promyelocytic leukemia HL-60 and histiocytic lymphoma U937 cell lines were cultured in the RPMI-1640 medium supplemented with a 10% calf fetal serum and antibiotics in 5% CO_2_ atmosphere at 37 °C. Cells were obtained from European collection of authenticated cell cultures (ECACC; Salisbury, UK)

The cell density and viability were measured using automatic analyzer Vi-CELL (Beckman Coulter, Brea, CA, USA). The Vi-CELL determines cell viability utilizing the trypan blue dye exclusion method [29].

### 4.2. Chemicals and Cell Treatment

NAC and thiourea (TU) purchased from Sigma-Aldrich (St. Louis, MO, USA) were dissolved in distilled water. Superoxide dismutase (*SOD*) from bovine erythrocytes and catalase (*CAT*) from bovine liver were dissolved in 25 mM Tris/HCl buffer, pH = 7.3. Cell treatment was done using 100–200 units of *SOD* per ml and 100 units of *CAT* per ml. Both enzymes were purchased from Sigma-Aldrich (St. Louis, MO, USA). 4-Aminobenzoic acid hydrazide (ABH), a myeloperoxidase inhibitor, obtained from Cayman Chemical (Ann Arbor, MI, USA) and VAS2870, a cell-permeable *NOX* inhibitor, obtained from Sigma-Aldrich (St. Louis, MO, USA), were dissolved in dimethyl sulfoxide (DMSO). The final concentration of DMSO in the culture medium did not exceed 0.1%.

Optional acidification of the growth medium with the highest concentrations of NAC (2 and 4 mM) was neutralized by the addition of sterile NaHCO_3_ solution (7.5%; Sigma-Aldrich, St. Louis, MO, USA).

### 4.3. Measurement of ROS Production

Intracellular ROS were monitored using fluorescent probe 2′,7′-dichlorodihydrofluorescein diacetate (H_2_DCF-DA; Molecular Probe, Leiden, The Netherlands). Originally, H_2_DCF-DA was used to detect of H_2_O_2_ [30].

Cells were stained with 5 μM H_2_DCF-DA for 30 min in growth medium in the dark at 37 °C. The fluorescence of deacetylated and oxidized DCF was analyzed using the flow cytometry Cytomics FC 500 System (Beckman Coulter) with argon laser excitation at 488 nm and emission at 525 nm [9]. At least 10,000 cells in each sample were analyzed.

### 4.4. mRNA Expression Profiling

Total RNA from cell stored in TRIzol was isolated using Direct-zol™ RNA Miniprep Kit (Zymo Research, Irvine, CA, USA) and transcribed using Transcriptor First Strand cDNA Synthesis Kit with anchored dT primers (Roche Applied Science, Indianopolis, IN, USA) according to the manufacturers’ recommendations. Quantitative RT-PCR was performed using TaqMan assays for *NOX3* (Hs01098883_m1), *NOX4* (Hs01379108_m1), *GPX2* (Hs01591589_m1, all Thermo Fisher Scientific, MA, USA), *GPX3* (Hs01078668_m1) using Platinum II Taq HotStart DNA polymerase (Thermo Fisher Scientific, Waltham, MA, USA) as reported previously [31]. Expression of *NOX1*, *NOX2*, *SOD1*, *SOD2*, *SOD3*, *CAT*, *GPX1*, *GPX4*, and *MPO* genes were assessed using the FastStart SYBR Green Master (Roche Applied Sciences, Indianopolis, IN, USA) as reported previously [32]; for primer sequences, see Table 1 (Integrated DNA Technologies, Coralville, IA, USA). A human universal reference RNA (Stratagene, La Jolla, CA, USA) was used as a calibrator and target gene expression was normalized to the housekeeping gene *PSMB2* [33]. RT-PCR was performed on a Rotor-Gene Q real-time PCR system (Qiagen, Germantown, MD, USA), relative mRNA expression levels were calculated by the second derivative method (Rotor-Gene Software 6.1.81, Qiagen, Germantown, MD, USA), as described previously [31,32,33].

### 4.5. Analysis of Cell Cycle and Apoptotic Cells

We adopted the protocol published by elsewhere [34]. Briefly, cells were harvested, washed in PBS, and then stained for 30 min in PBS containing 0.1% Triton X-100, propidium iodide, (PI; 10 µg/mL), RNase A (100 µg/m) prior to flow-cytometric analysis on a Cytomics FC 500 System (Beckman Coulter). The data were analyzed using MultiCycle software (P.S. Rabinovitch, University of Washington, Seattle, WA, USA).

This protocol enables analysis of the cell cycle progression as well as the identification of apoptotic cells. Activation of endonucleases in apoptotic cells leads to a reduction in DNA content because hypotonic buffers containing a non-ionic detergent have the ability to extract cleaved low molecular weight DNA out of the cells into solution. Therefore, apoptotic nuclei appeared as a broad hypodiploid DNA peak, which can be easily discriminated from live cycling diploid cells [34,35].

### 4.6. Morphological Analysis of Apoptosis

Cells were fixed and stained with 4′,6-diamidino-2-phenylindole (DAPI), as described previously [36]. Nuclear morphology was examined using an Olympus BX60 (Olympus, Hamburg, Germany) fluorescence microscope (magnification 400×, excitation filter band 360–370 nm, emission filter band 420–460 nm).

### 4.7. Measurement of Caspase Enzymatic Activity in Cell Extracts

Caspase-3/7 (DEVDase) and caspase-9 (LEHDase) enzyme activities were determined using fluorescent substrates Ac-DEVD-AMC, and Ac-LEHD-AFC, respectively [19,37]. Briefly, cells washed in ice-cold PBS were lysed in caspase extraction buffer [50 mM HEPES, pH 7.4, 1 mM EDTA, 1 mM EGTA, 1 mM DTT, 0.2% Chaps, and proteinase inhibitor cocktail (Sigma-Aldrich, St. Louis, MO, USA)] for 20 min at 4 °C. Cell extracts were clarified by centrifugation (30,000× *g*, 10 min at 4 °C). The assay was carried out in 96-well plates. Aliquots of cell lysates (50 μg of total protein) were mixed with assay buffer (50 mM PIPES/KOH, 2 mM EGTA, 2 mM MgCl_2_, and 5 mM DTT, pH 7.4, and 6.6 for caspase-3/7 and caspase-9, respectively) in a final volume of 200 µL. Reactions were initiated by adding substrates, Ac-DEVD-AMC (50 µM) and Ac-LEHD-AFC (50 µM). After 30 min, incubation at 37 °C fluorescence was monitored at excitation, and emission wavelengths of 380 and 445 for caspase-3/7, and 400 and 500 nm for caspase-9. A non-specific substrate cleavage, which was determined as residual substrate cleavage in the presence of 1 µM Ac-DEVD-CHO (for caspase-3/7) and 1 µM Ac-LEHD-CHO (for caspase-9), was subtracted from every measured value of activity.

### 4.8. Statistical Analysis

Data are reported as the means ± S.E. (standard error). Statistical significance was determined by the Student’s *t*-test. *p* values equal to or less than 0.05/0.01 were considered significant/very significant. Statistical analyses were performed using the SigmaPlot 11.0 software package (Systat Software Inc., San Jose, CA, USA).

## 5. Conclusions

In conclusion, NAC can induce massive production of O_2_•^−^ in both, promyelocytic leukemia HL-60 and histiocytic lymphoma U937 cell lines. O_2_•^−^ itself does not induce the cell death. Its conversion to hydrogen peroxide by *SOD* is a necessary precondition for this. While peroxide formation sufficient for cell death induction is catalyzed by endogenous expression of *SOD1* and *SOD3* in HL-60 cells, the addition of extracellular *SOD* is required to induce cell death in U937 cells. Conversion of H_2_O_2_ to ClO^−^ by endogenous *MPO* further contributes to the induction of cell death, but only in HL-60 cells. In contrast, extracellularly added *CAT* prevents cell death in both cell lines. The mode of cell death in response to NAC treatment bears apoptotic and apoptotic-like features in HL-60 and U937 cells.

## Figures and Tables

**Table 1:** Primer sequences used for qRT-PCR.

Gene	Forward Primer (5′ to 3′)	Reverse Primer (5′ to 3′)
*NOX1*	AAGGATCCTCCGGTTTTACC	TTTGGATGGGTGCATAACAA
*NOX2*	GGCTTCCTCAGCTACAACATCT	GTGCACAGCAAAGTGATTGG
*SOD1*	TCATCAATTTCGAGCAGAAGG	GCAGGCCTTCAGTCAGTCC
*SOD2*	GCACTAGCAGCATGTTGAGC	CCGTAGTCGTAGGGCAGGT
*SOD3*	GGTGCAGCTCTCTTTTCAGG	AACACAGTAGCGCCAGCAT
*CAT*	TCATCAGGGATCCCATATTGTT	CCTTCAGATGTGTCTGAGGATTT
*GPX1*	CAACCAGTTTGGGCATCAG	TCTCGAAGAGCATGAAGTTGG
*GPX4*	TTCCCGTGTAACCAGTTCG	CGGCGAACTCTTTGATCTCT
*MPO*	CGTCAACTGCGAGACCAG	GTCATTGGGCGGGATCTT

## Data Availability

The data presented in this study are available upon request from the corresponding author.
